# Monitoring hunted species of cultural significance: Estimates of trends, population sizes and harvesting rates of flying-fox (*Pteropus sp*.) in New Caledonia

**DOI:** 10.1371/journal.pone.0224466

**Published:** 2019-12-31

**Authors:** Malik Oedin, Fabrice Brescia, Mélanie Boissenin, Eric Vidal, Jean-Jérôme Cassan, Jean-Claude Hurlin, Alexandre Millon

**Affiliations:** 1 Institut Agronomique Néo-Calédonien (IAC), Equipe ARBOREAL (AgricultuRe BiOdiveRsité Et vALorisation) BP, Païta, Nouvelle-Calédonie; 2 Institut Méditerranéen de Biodiversité et d’Ecologie marine et continentale (IMBE), Aix Marseille Université, CNRS, IRD, Avignon Université, Centre IRD Nouméa, Nouvelle-Calédonie; 3 UMR Entropie (IRD, Université de La Réunion, CNRS), Labex-Corail, Institut de Recherche pour le Développement (IRD), Nouvelle-Calédonie; 4 Province Nord, Direction du Développement Economique et de l’Environnement, Service impact environnemental et conservation (DDEE), Service impact environnemental & conservation, Koné, Nouvelle-Calédonie; 5 Aix Marseille Université, CNRS, IRD, Avignon Université, Institut Méditerranéen de Biodiversité et d’Ecologie marine et continentale (IMBE), Technopôle Arbois-Méditerranée, Aix-en-Provence, France; University of Sydney, AUSTRALIA

## Abstract

Assessing population trends and their underlying factors is critical to propose efficient conservation actions. This assessment can be particularly challenging when dealing with highly mobile, shy and nocturnal animals such as flying-foxes. Here we investigated the dynamics of hunted populations of *Pteropus ornatus* and *P*. *tonganus* in the Northern Province of New Caledonia. First, an ethno-ecological survey involving 219 local experts identified 494 flying-fox roosts. Current status was assessed for 379 of them, among which 125 were no longer occupied, representing a loss of 33% over ca. 40 years. Second, species-specific counts conducted at 35 roosts, and a sample of animals killed by hunters, revealed that the endemic species, *P*. *ornatus*, was dominant (68.5%). Between 2010 and 2016, 30 roosts were counted annually during the pre-parturition period. Roosts size averaged 1,425 ± 2,151 individuals (*N* = 180 counts) and showed high among-year variations (roost-specific CV = 37–162%). If we recorded significant inter-annual variation, we did not detect a significant decline over the 7-yr period, although one roost went possibly extinct. Population size of the two species combined was estimated at 338,000−859,000 individuals distributed over ca. 400 roosts in the Northern Province. Flying-foxes are popular game species and constitute traditional food for all communities of New Caledonia. Annual bags derived from a food survey allowed us to estimate harvesting rates at 5–14%. Such a level of harvesting for species with a ‘slow’ demography, the occurrence of poaching and illegal trade, suggest the current species use might not be sustainable and further investigations are critically needed.

## Introduction

In the current context of rapid loss of biodiversity [1], identifying wildlife populations at risk and implementing evidence-based conservation actions are priorities. Habitat loss and degradation, invasive species and overharvesting are the three main threats to biodiversity to date, with climate change possibly magnifying such effects [2–8]. When species are suffering from multiple threats simultaneously, it becomes urgent to transcend surveillance monitoring and implement adaptive management strategies [9].

Of the 65 taxa of flying-foxes (fruit bats of the genus *Pteropus*), those occurring on islands (53 species) are among those species facing multi-faceted threats [10–13]. The conservation of flying-foxes in tropical islands is of paramount importance as they are key species in the functioning of insular ecosystems [13,14], providing essential ecological services such as plant pollination and seed dispersal [15–17]. Most flying-fox species are hunted, legally or not, including species with vulnerable conservation status or worse (at least 49 species; [11,13]. Overexploitation and habitat loss have already led to the extinction of four species of flying-foxes and brought others to the verge of extinction, such as *Pteropus rodricensis*, *P*. *aruensis* or *P*. *pselaphon* [11]. Their habit of forming large camps for roosting during the day, gathering hundreds to thousands of individuals, makes flying-foxes particularly sensitive to hunting, with negative effects of disturbance of a large number of animals adding to mortality [10,12,18–20].

Over millennia, flying-foxes have co-existed with humans in South West Pacific islands and have, in many instances, become key elements to Melanesian cultures as totem animals, ceremonial food, and element of the manufacturing of sacred items (traditional currency or weapon) as well as medicine [21–24]. Yet, the spreading of firearms and the increase of recreational hunting by other communities currently challenge the concept of sustainable harvesting in many areas [11,13]. Ensuring the sustainability of such complex socio-ecological systems, involving species facing multi-faceted threats, is a major challenge ahead of conservation biologists [9,25,26]. However, very few perennial monitoring programs of flying-foxes dynamics are on-going (Australia, Madagascar and New Caledonia; [10,27,28], thereby preventing the assessment of population dynamics and the onset of effective conservation actions. This situation is partly due to the notorious difficulty of monitoring highly mobile, gregarious and nocturnal organisms such as flying-foxes [29]. Count data are then associated with high spatial and temporal variance, thus reducing the ability to detect significant population trends.

Bats are the only terrestrial native mammals of the New Caledonian archipelago (South Pacific, Melanesia), and this biodiversity hotspot is hosting four flying-fox species, three of them being endemic [30]. The two largest species, the endemic ornate flying-fox *Pteropus ornatus* (Gray, 1870) and the native Pacific flying-fox *Pteropus tonganus* (MacGillivray, 1960), are hunted for food by all communities inhabiting the island. Furthermore, flying-foxes play a central role in the culture of the Melanesian community (Kanak) [31,32] especially during the yam celebration, the main social event of the Kanak culture [33,34]. Flying-foxes have a relatively ‘slow’ demography (maximum one young per female per year) and their resilience to the current harvesting pressure in New Caledonia is unknown.

Here, in an attempt to provide first evidences about population dynamics and harvesting rates of flying-foxes in New Caledonia, we took advantage of expert interviews to gather local knowledge about location of diurnal roosts since the early 1970’s. This survey has led to a large-scale roost inventory in the largest region of New Caledonia, the Northern Province, to assess long-term persistence of flying-fox roosts as well as their specific composition. In addition, we analysed data from a sample of roosts that has been monitored annually in order to provide estimates for population size and trend (2010–2016). Combining such data to a recent household survey on food consumption, we derived harvesting rates for *Pteropus ornatus* and *P*. *tonganus* combined. Finally, we discussed potential actions to be undertaken to ensure the long-term sustainability of flying-fox hunting in New Caledonia.

## Methods

### Model species

The ornate flying-fox *Pteropus ornatus* is one of the three species endemic to New Caledonia and considered as Vulnerable (IUCN, 2018). The Pacific flying-fox *P*. *tonganus* is widespread over South Pacific islands and considered as Least Concern [11]. Both species are large-bodied fruit bats with a wing-span of 100–110 cm and weight averaging 669 ± 69 g (*N* = 58) and 629 ± 73 g (*N* = 8) for adult *P*. *ornatus* and *P*. *tonganus*, respectively [27,35,36]. These two species occur over the whole New Caledonia and share ecological requirements to a large extent. Diurnal roosts typically gather the two species on the same trees.

*P*. *ornatus* and *P*. *tonganus* show similar reproductive schedules with mating occurring from March to May. The gestation period lasting about six months, most births are observed from mid-September to mid-November. Lactation spans until March/April, such that breeding females are involved in reproduction all year long (S1 Fig). Females can breed from the age of three years and raise one offspring per year (occurrence of twins being exceptionally recorded; [20,31,36]).

### Study area, expert interviews, roost inventory, count methods & annual bags

The surveys took place in the Northern Province of New Caledonia mainland (21.1° S, 164.9° E; 9583 km^2^ i.e. 52% of the territory). It is composed of 203 Melanesian tribes (Kanak) in 17 municipalities and totalling 18.8% (50,487 people including 35.578 Kanaks) of the whole New Caledonian population [37]. A roost inventory was based on field records and interviews of local experts including hunters, naturalists, nature guides, wildlife rangers and land owners, from Kanak and other communities. The survey was completed between 2006 and 2009 and covered 87% of the Northern Province area. The experts were identified according to their local reputation regarding flying-fox knowledge. The interviews were conducted to collect expert knowledge about roost locations and their history (current status, disappearance or recent appearance). A total of 370 persons were interviewed among which 219 provided relevant information.

Following this survey, a sample of 35 roosts was selected in 2008, spread over the whole Northern Province and encompassing the whole range of roost size (35–4,000 bats) to estimate the relative proportion of the two species. Direct observations were made at large distance (>100m), with a 20×60 telescope to avoid disturbance. This study was approved by the environmental service of the Northern Province of New Caledonia. We got permission from all land owners in case roosts were located on private land. All detected individuals were identified as *P*. *ornatus* or *P*. *tonganus* based upon coat colour and the shape of the golden breast patch. The specific proportion obtained at each roost was then averaged across the 35 roosts and weighted by roost size (estimated with the same method as described below). To ascertain this specific proportion, we used an additional source of data based on a sample of animals killed by hunters. The mean proportion between these two independent datasets was used to derive the specific proportion and population size.

**Fig 1:**
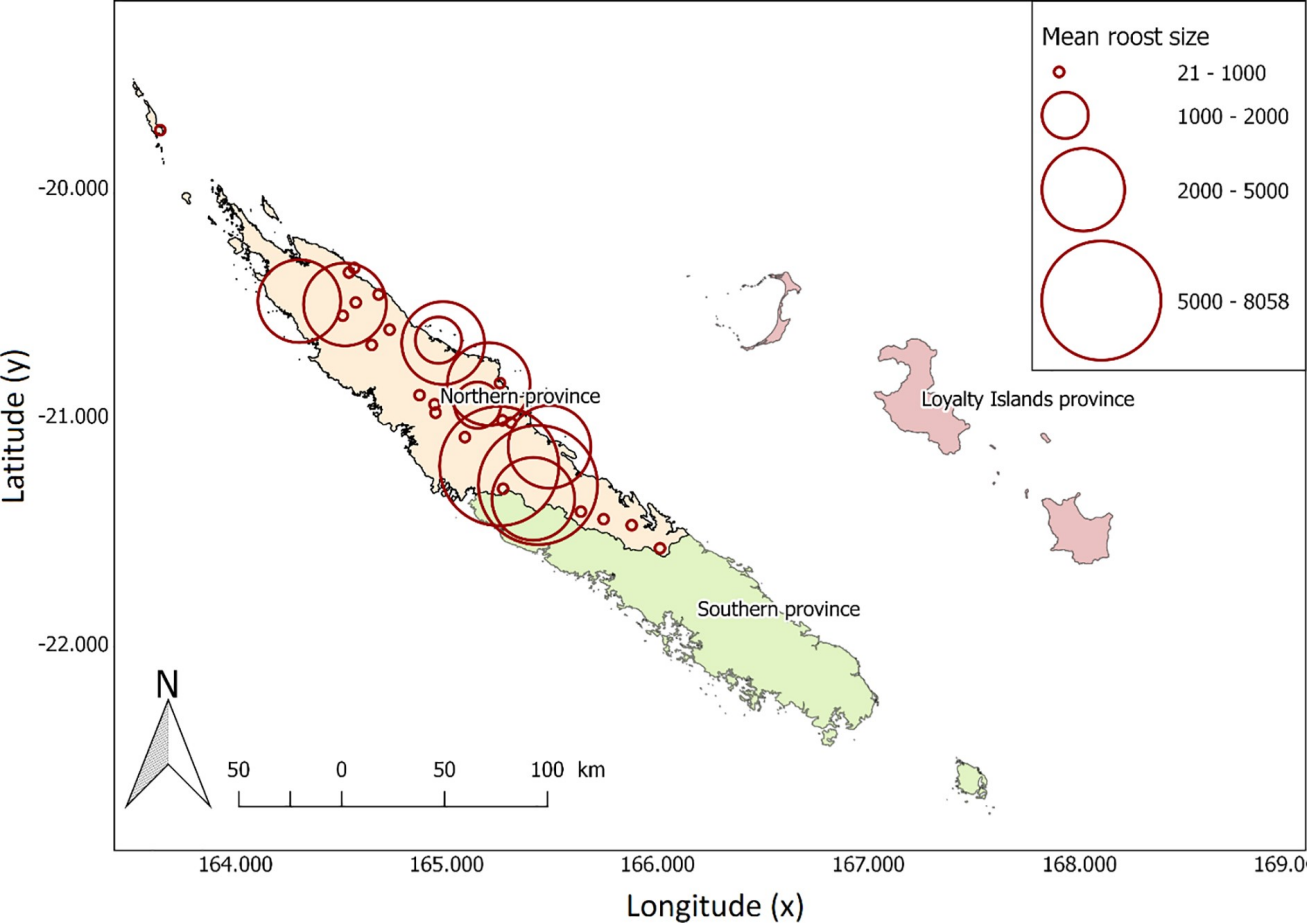
Locations of the 30 surveyed roosts of ornate and Pacific flying-foxes in the Northern Province of New Caledonia. The size of the circles is proportional to the average number of flying-foxes counted at roosts over the 2010–2016 survey period (Geographic coordinate systems: *RGNC* 1991 / Lambert New Caledonia).

**Fig 2:**
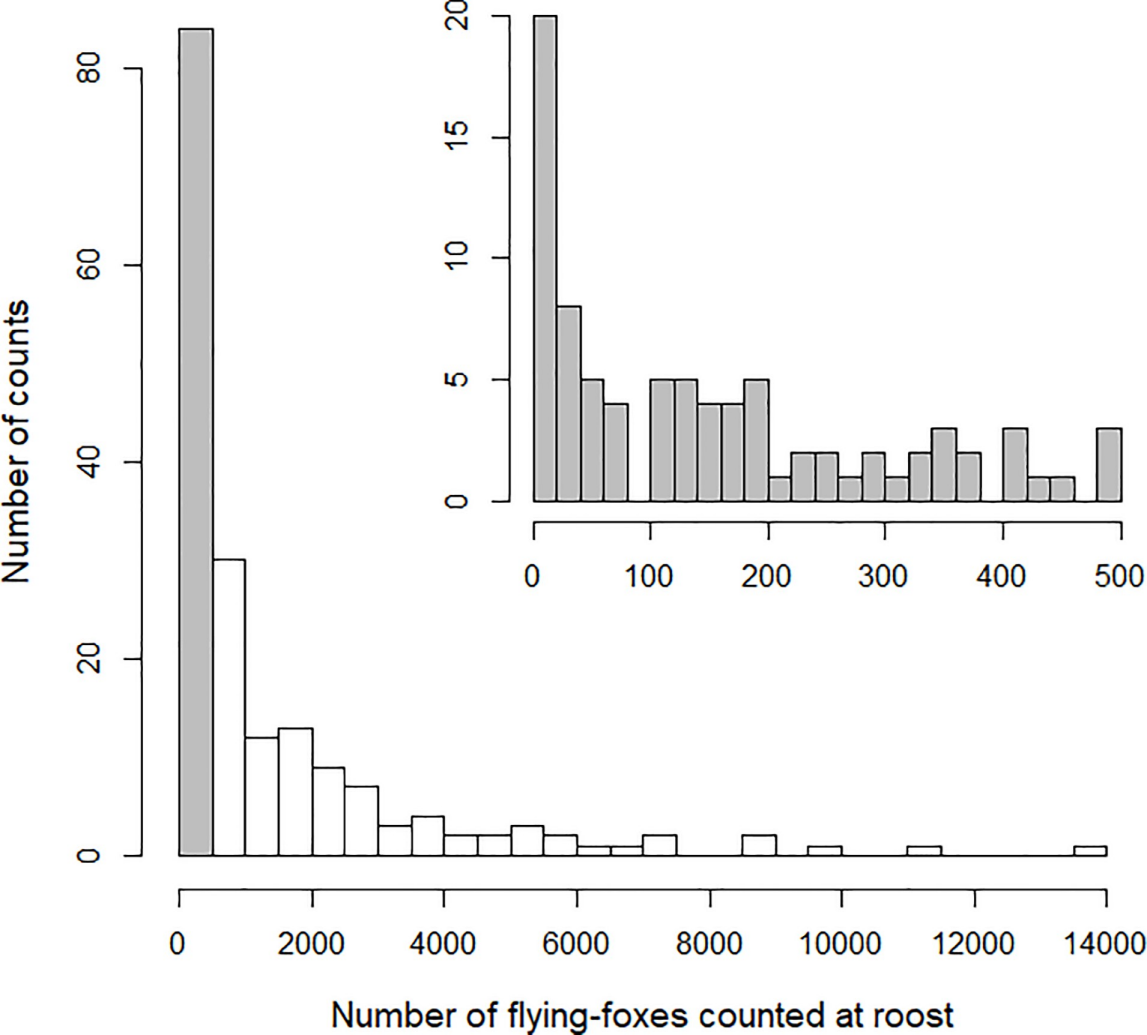
Distribution of the 180 counts made on the sample of 30 flying-foxes roosts of ornate and Pacific flying-foxes in the Northern Province of New Caledonia between 2010 and 2016. Counts of 0 were made in 9 occasions on four different roosts. The inserted histogram further details the distribution of the first interval (0–499 individuals; grey bar).

Between 2010 and 2016, thirty day roosts, for which the location allowed us to carry out proper counts, were chosen to conduct fly-out counts. This selection gathered roosts of all sizes (roost mean counts ranging from 21 ± 27 to 5328 ± 2893; see Fig 1 and Fig 2) as an attempt to obtain a representative view of the roosts occurring in the Northern Province. However, so as to ensure repeated surveys could be carried out over the long-term given the involvement of local people, roots were also selected for their relative accessibility. As a consequence, the roots counted were closer to tribes than expected by chance (log-log model; *β* = −0.24 ± 0.06, *P* <0.001). We found no correlation between roost abundance (average across years) and distance to tribe (*r* = 0.004, *P* = 0.99), although we acknowledge this correlation is calculated on a truncated distribution of distance (S2 Fig).

Counts took place annually in September, at the start of the parturition period, characterized by important gathering for both species [36,38,39]. Of the 30 roosts surveyed, 16 have been counted the seven years of the study (average number of counts per roost: 5 ± 2, 180 counts in total). Flying-foxes were counted at dusk (from 1 hour before sunset until no bat was detected for a period of 15 minutes, usually corresponding to 45 minutes after sunset) when leaving the roost for foraging (see also [10,40–42]). The two species cannot be distinguished during fly-out and counts refer thus to total abundance. Fly-out counts realised from a vantage point were preferred over counts realised below the roost [38–42], despite this technique has sometimes been judged as less accurate [43–45]. However, we believe this method was the most appropriate for counting flying-foxes in New Caledonia as: i) roosts are located in dense tropical forests where the canopy often hides a large proportion of animals, ii) flying-foxes are highly sensitive to disturbance when roosting, possibly because of intensive hunting, and do not tolerate observers walking close to roosting trees (typically <300m) and iii) maximal roost size recorded in New Caledonia (19,000 individuals) did not exceed observers’ counting capability [38,39]. Only one roost was counted during daytime from a vantage point because of the exceptional visibility of the animals within the trees. Counts were systematically made by a pair of observers located on the same vantage point, each of them being responsible for counting half of the skyline. A pilot study has been conducted in April 2010 on 21 roosts so as to train 27 observers and identify adequate vantage points in order to maximise count accuracy. To reduce possible bias due to strong daily variations (e.g. because of adverse weather), counts were repeated over three consecutive days and the maximum number was selected for the analyses. For counts performed for one or two days only (*N* = 16), we applied a correction based on the average percentage of flying-foxes missed estimated from counts over three days (one count, N* = N + 28%; two counts, N* = N_max_ + 18%). Data are available as online supplementary material (S1 Fig and S1 File).

The number of flying-foxes harvested over a year was estimated from a sociological survey conducted by Quid Novi for the New Caledonian Government, on household consumption, including natural resources, based on a sample of 200 households per month from all New Caledonian communities in each Province in 2016 and 2017 (Direction de l’Environnement Province Sud, *pers*. *comm*.).

### Data analyses

We tested for the occurrence of a linear trend in total flying-fox abundance over time using Generalized Linear Mixed Models (GLMM) using a negative binomial distribution of error. The negative binomial distribution was preferred over a Poisson distribution because of the large over-dispersion observed in counts. Successive annual counts at the same roost cannot be considered as fully independent statistical units. To account for this, we added roost identity as random factor and a first-order autoregressive correlation structure (AR1) to model residuals [46]. Spatial proximity among roosts can also affect the hypothesis of data independence so we assessed spatial autocorrelation in model residuals. No pattern was detected, as confirmed by the use of *testSpatialAutocorrelation* function, *blemco* package. The adequate random structure was selected according to a likelihood ratio test between umbrella models with and without random structure [46]. As a final step, we plotted the distribution of standardised residuals according to each covariate retained in the best model to verify homoscedasticity. We further investigated the potential inter-annual non-linear variation in flying-fox abundance using Generalized Additive Mixed Models (GAMM), with year as a smooth term and other model specifications as above.

We estimated the total population size of *P*. *ornatus* and *P*. *tonganus* in the Northern Province by randomly selecting 30 counts out of the 180 roost counts available using a bootstrap procedure with replacement (1,000,000 iterations). The median population size obtained was then multiplied by the number of roosts recorded in the Northern Province, assuming 1) the roosts left unchecked had a probability of persistence equal to the sample of roosts that have been checked and 2) the fraction of the study area not sampled (13%) host similar roost densities. This procedure assumes all roosts have been detected by local stakeholders in the sampled area and may therefore provide a conservative figure.

Analyses were carried out using R 3.4.0 [47]. Models were run using the function *glmer*.*nb* from the package *lme4* for GLMMs, the function *gamm* from the package *mgcv* for GAMMs and the function *lme* from the package *nlme* for LMMs. Residual dispersion was assessed using the package *DHARMa*.

## Results

### Roost persistence based according to expert local knowledge

The information gathered from 219 experts allowed us to identify 494 flying-fox roosts across the Northern Province of New Caledonia. A total of 379 roosts were checked in the field (115 left unchecked due to budget limitation), among which 254 were still active and 125 were no longer found occupied despite intensive research. This represents a loss of 33%. The time scale of this loss was defined following experts’ answers, between 30 and 40 years (prior to 2009). No expert notified the appearance of a roost over his/her lifetime.

### Roost specific composition, total population size and harvesting rate

Over the 35 roosts surveyed for estimating specific composition, species determination was possible for 19,997 individuals (mean number of individuals per roost: *N* = 767 ± 960). The proportion of the endemic *P*. *ornatus* averaged 60% ± 25 (range: 2–100%) and was not significantly correlated with roost size (*r* = −0.17, *P* = 0.33). The proportion of *P*. *ornatus* derived from animals killed by hunters was higher (77%, *N* = 155). The specific proportion between these two datasets averaged 68.5%.

Flying-foxes roost size averaged 1,425 ± 2,151 individuals (median = 559, range = 0−12,504; *N* = 180 counts on 30 roosts; Figs 1 & 2). Considering solely the 16 roosts surveyed over the 7 years of the study, counts averaged 1,625 ± 2,552 individuals (median = 554, *N* = 112 counts).

Assuming our roost sample was representative for the population occurring in the Northern Province, the median population size of two species combined was estimated at 563,000 flying-foxes (90% Confidence Interval: [338,000−859,000]) distributed across an estimated 399 roosts. Considering the estimated specific composition, population size was estimated at 232,000−588,000 and 106,000−271,000 for *P*. *ornatus* and *P*. *tonganus*, respectively. With an annual number of flying-foxes killed by hunters over the Northern Province estimated at 45,724, the median harvesting rate reached 9.4% [5.3−13.5] for both species combined.

### Temporal variation in flying-fox abundance

Among the 30 roosts monitored with annual fly-out counts, three roosts showed temporary disappearance (one year) over the seven years of the study, and another roost hosting 481 ± 374 individuals between 2010 and 2012, fell down to 5 individuals in 2013 and none over the last 3 years.

**Fig 3:**
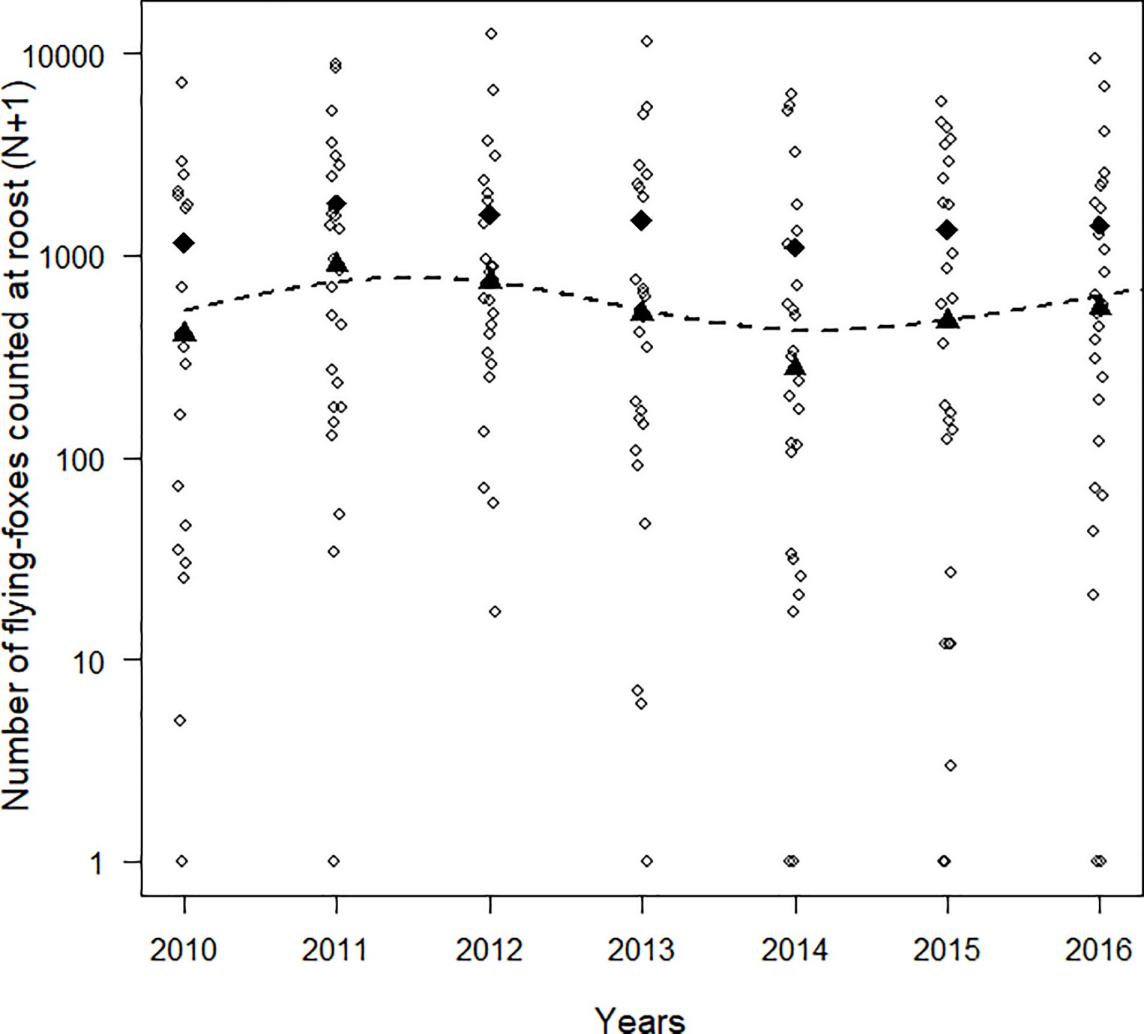
Combined population trends of ornate and Pacific flying-foxes in the Northern Province of New Caledonia from a sample of 30 roosts counted between 2010 and 2016. Annual average and median values are depicted by diamond shapes and triangles respectively. The dotted line refers to the mean predicted value from the GAMM analysis. Note the log_10_-transformed y-axis.

Roost counts showed a strong among-year variation with an average coefficient of variation of 85% (range among roosts: 37–162%, *N* = 30 roots; Fig 2, S3 Fig). Overall abundance of flying-foxes showed no significant temporal trend between 2010 and 2016 (GLMM, *β* = −0.075 ± 0.088 on log scale, *P* = 0.4). The GAMM analysis revealed a significant non-linear variation of the number of flying-foxes over time with a slight increase from 2010 to 2011, followed by a decline until 2014 and another slight increase until 2016 (estimated degree of freedom = 3.52, *F* = 2.83, *P* = 0.02; Fig 3).

## Discussion

A third of the 379 diurnal roosts of flying-foxes for which we collected a precise location from local knowledge in the Northern Province of New Caledonia seems to have disappeared over 30–40 years (1970’s-2000’s). Diurnal roosts typically hosted the two species, *Pteropus tonganus* and *P*. *ornatus*, with a higher proportion of the latter (60%), endemic to New Caledonia, and gathered an average of 1,425 ± 2,151 individuals. We estimated the total population size of these two species for the Northern Province at 338,000−859,000 flying-foxes distributed over ca. 400 roosts. The annual hunting bag by the different communities composing the Northern Province suggested an annual harvesting rate of 5.3−13.5%. A count survey realized between 2010 and 2016 on a sample of 30 roosts showed overall stability with slight inter-annual variations, although one roost went possibly extinct.

### Estimates of temporal trends from expert and count surveys

While the count survey revealed an apparent stability of flying-fox numbers between 2010 and 2016, evidence gathered from local experts seems to indicate an important loss of roosts over three to four decades. This apparent contradiction partly arose from the mismatch between survey periods (2010–2016 *vs*. 1969–2009) and lengths (7 *vs*. 30–40 years). Nevertheless, these results could be reconciled if we consider that one of the 30 roosts included in the count survey possibly disappeared over the seven-year period. The disappearance of a third of the roosts over 40 years is equivalent to an annual loss of 0.75%. Reported to a sample of 30 roosts over 7 years, we can expect a loss of 1.6 roosts.

Expert interviews allowed us to collect information on roosts locations, subsequently checked in the field, to estimate flying-fox population dynamics on large spatial and temporal scales, with a relatively limited amount of resources. Such an approach, however, is not free of biases. First, imprecise localisations gathered from experts or failure to detect roosts in the field may have led to the wrong record of a roost loss. If a small roost may indeed remain unnoticed during the day, observations from vantage points at dusk when flying-foxes are leaving their roost ensured a high detection probability. Second, roosts may have moved from one place to another over the long period of time considered here. Flying-foxes are well known for their high fidelity to roosting sites, even when subject to some level of disturbance [20,48–51]. However, we believe such data provide useful evidence for assessing the status of flying-foxes and, overall, it is unlikely the aforementioned biases can explain the observed loss. This provides an example of how much citizen science can be useful and informative when facing a knowledge gap with no historical record available [52–55].

Counts of animals typically suffer from high variance, especially when considering highly gregarious and mobile species such as flying-foxes [43–45]. The large variance observed here (CV of global average: 151%) certainly reduced the power to detect a significant trend over seven years. It is noteworthy, however, that a significant among-year variation was detected with the generalized additive modelling, suggesting our survey method might be adequate to monitor long-term trends of New Caledonian flying-foxes. In order to limit the impact of between-roost movements on estimates, we systematically conducted counts during the same, relatively restricted, period (September). Count accuracy can also be affected by meteorological conditions or directions used by flying-foxes when leaving the roost to join foraging areas. As an answer to this, we performed counts on three consecutive days in most instances. Movements among a set of connected roosts can also add noise to the data. These two factors, however, if they reduce the statistical power to detect a temporal trend, are unlikely to systematically bias our results. If our results suggest an apparent stability over the short-term, we have however to be cautious as important inter-annual variations may hide an actual decline.

### Estimates of population size and harvesting rate

Based upon our expert and roost count surveys, we estimated the flying-fox population size in Northern Province between 338,000 and 859,000 individuals distributed over ca. 400 roosts (*P*. *ornatus* and *tonganus* combined). Considering median estimates, population size reached 338,000 individuals for the endemic *P*. *ornatus* and 225,000 for *P*. *tonganus*.

We estimated harvesting rates by the different communities of the Northern Province of New Caledonia between 5.3 and 13.5%. Hunting of *P*. *ornatus* and *P*. *tonganus* is regulated by two globally similar provincial laws on mainland. The hunting period is restricted to 8–10 days in April (weekends only) with a daily quota of five animals per hunter. Hunting is strictly forbidden at <300m from roosting sites and after dusk. Such regulations, however, only slightly overlap with the schedule of traditional hunting by the Kanak community around Yam celebration (S1 Fig). The Yam celebration, the main social event of the Kanak community (105,000 persons in New Caledonia; [37]), includes in most tribes a traditional meal, typically including flying-foxes, and occurs each year between February and July (S1 Fig; [32,34,56]). This mismatch between regulation and main use is likely to encourage poaching activities, by all communities, especially given the relatively weak law enforcement.

In New Caledonia, flying-foxes are mostly hunted in flight with fire arms shortly after they leave diurnal roosts for foraging at dusk and at night. Crippling losses inherent to this hunting technique may significantly increase mortality rates due to hunting as 1) a proportion of bats are only wounded and die shortly after and 2) hunters recover only a fraction of the animals they killed, as finding a flying-fox in the dense tropical vegetation with low luminosity is difficult. In addition, gunshots used for hunting flying-foxes were regularly recorded during our count survey in September (1 ± 6 per count, range: 0−100; *N* = 441 roost counts), suggesting poaching actually occurs throughout the year. Poaching is indeed supported by an illegal trade with one flying-fox worth 1000–7000 XPF on black market (10–70 USD; IAC, *unpub*. *data*). Altogether, further data are needed to more precisely estimate harvesting rates and to investigate whether current hunting bags are sustainable [57]. The relatively ‘slow’ demography of *Pteropus* species (delayed maturity of females at 3-yr-old, maximum of one offspring per year, maximum age in captivity up to 30 yr-old [58], mean age in the wild of 7–15 years [13,59,60]) suggests their populations can only sustain a low to moderate harvesting rate [10,12,18–20,61]. Current regulations are ill-adapted to the use of flying-foxes by most stakeholders, a context probably promoting poaching. An adaptive management strategy including open discussions between stakeholders about the period of hunting, the onset of a maximal quota, based on our results, and the systematic tagging of killed animals might improve the situation by reducing poaching, providing local specificities are appropriately accounted for.

Bushmeat trade has been widely recognized as having a strong impact on animal populations [62]. Regarding flying-foxes, hunting has been probably the main factor leading to the extinction of *P*. *tokudae* on Guam [11,13,20,63,64]. Poaching can occur directly at roosts, leading to a large number of animals killed in addition to disturbance, the latter being identified as one of the main factor causing roost desertion [20,51,65]. In Niue Island (South Pacific), flying-foxes abandoned their roost, following intense disturbance by hunting, and returned only after 5–10 years [66]. MacKinnon et al. (2003) reported that 27 of 154 *P*. *rufus* roosts surveyed in Madagascar were abandoned over 10 years, mainly as a result of hunting and destruction of roost trees [67]. Flying-foxes typically show high roost fidelity. Nevertheless, high level of perturbation may force them to desert and cause major disruption to populations [20,48,49,51,68]. The loss of flying-fox roosts we documented here may, at least partially, be the consequence of direct but also indirect (disturbance) effects of hunting.

### *Pteropus* species in New Caledonia face multiple threats

Current knowledge highlights a negative impact of various environmental factors on flying-fox populations worldwide: habitat destruction, hunting, extreme climatic events (cyclone, heat wave) and invasive species [19,20,28,65,69]. In New Caledonia, human activities, such as farming, mining (nickel extraction) and urbanisation [70,71] have reduced the extent, and increased the fragmentation, of wet primary forests by 70% [30]. Wet forests constitute the main habitat of *Pteropus* species for both roosting and foraging [20,72,73]. New Caledonia is also currently subject to an increase of fires leading to further forest loss [74–77]. Moreover, the archipelago is exposed to cyclones which may affect flying-foxes directly (injury, mortality) but also indirectly by damaging forests and temporarily depleting fruit and flower resources. Cyclones entail cascading effects by forcing them to feed near human settlements, sometimes directly on the ground exposing them to poaching and predation [28,78,79].

Culling programs have also been undertaken following perceived bat-human conflicts regarding fruit production and risk of disease transmissions to humans, notably in Australia and Mauritius [80–82]. New Caledonian flying foxes are also potentially exposed to this risk as highlighted by the killing of 39 flying-foxes in 2015 in the Southern province to estimate the prevalence of Nipah virus in the wild following detections in captive individuals (Direction des Affaires Vétérinaires, Alimentaires et Rurales de Nouvelle-Calédonie, *pers*. *comm*.).

Finally, a previously unsuspected threat has been recently identified with evidence of predation or competition for food resources by invasive species such as cats, dogs, rats or ants [83–85]. In New Caledonia, flying-foxes are commonly found in cat scats all-year round, and in most of forest habitats, but both the way predation is achieved and its impact on populations remains to be assessed [86].

## Conclusions and recommendations

Here we provided first estimates of population size and dynamics of two flying-fox species over the largest province of the New Caledonian mainland. There, flying-foxes play a central role in forest ecosystem functioning and are major game species for the different communities composing New Caledonia. Evidences for intensive hunting and poaching practices suggest annual bags are substantial and further data on flying-fox demography (anthropogenic mortality, fecundity but also movements) are critically needed to assess the long-term sustainability of the species use and, if necessary, to set new hunting regulations.

## Supporting information

S1 Fig Annual biological cycle of *Pteropus ornatus* and *P*. *tonganus* in New Caledonia and temporal extent of the legal hunting period and of the Yam celebration. (TIF)

S2 Fig Distance to (a) the nearest town and (b) the nearest tribe according to whether the flying-fox roosts were part of the set of roosts selected for fly-out counts (counted: yes/no). (c) Correlation between the average size of roost and distance to the nearest tribe (both variables are log-10 transformed; *r* = 0.004, *P* = 0.99).
(TIF)

S3 Fig Time-series of flying-fox counts (*Pteropus ornatus* and *P*. *tonganus* combined) for each of the 30 roosts surveyed in the Northern Province of New Caledonia between 2010 and 2016. Counts were made in September during the pre-parturition period over one to three consecutive days (maximum counts used for analyses with correction if less than three counts were performed, see Methods). Red dots indicated counts of 0.
(TIF)

S1 File Time-series of flying-fox counts (*Pteropus ornatus* and *P*. *tonganus* combined) for each of the 30 roosts surveyed in the Northern Province of New Caledonia between 2010 and 2016. Counts were made in September during the pre-parturition period over one to three consecutive days (count_repetition).
(XLSX)
