# Supplementary figures and images for: Monitoring hunted species of cultural significance: Estimates of trends, population sizes and harvesting rates of flying-fox (Pteropus sp.) in New Caledonia
Source: PLoS One. 2019 Dec 31;14(12):e0224466. doi: 10.1371/journal.pone.0224466 (PMC6938311; doi:10.1371/journal.pone.0224466)

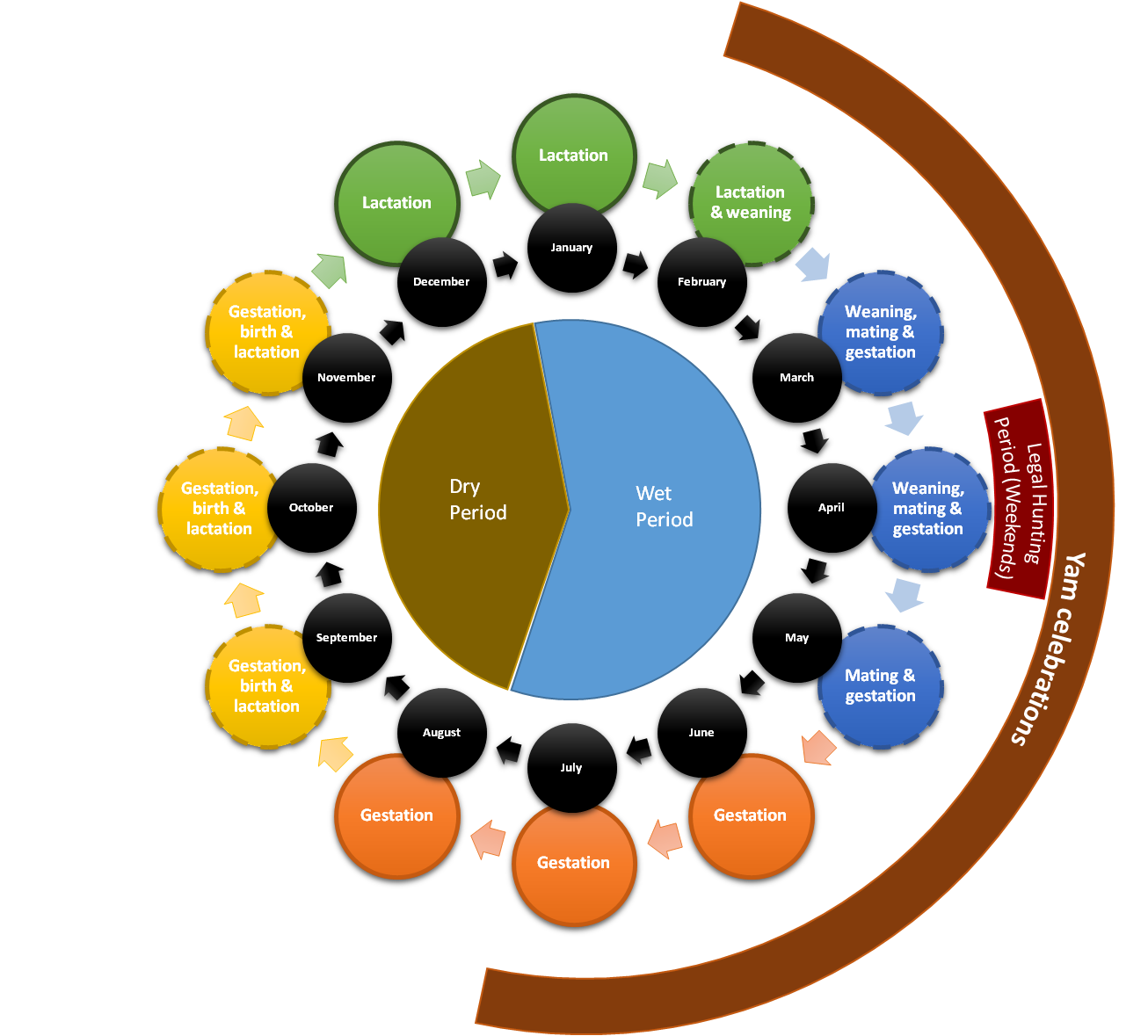

Supplement: S1 Fig — (TIF) [file pone.0224466.s001.tif]

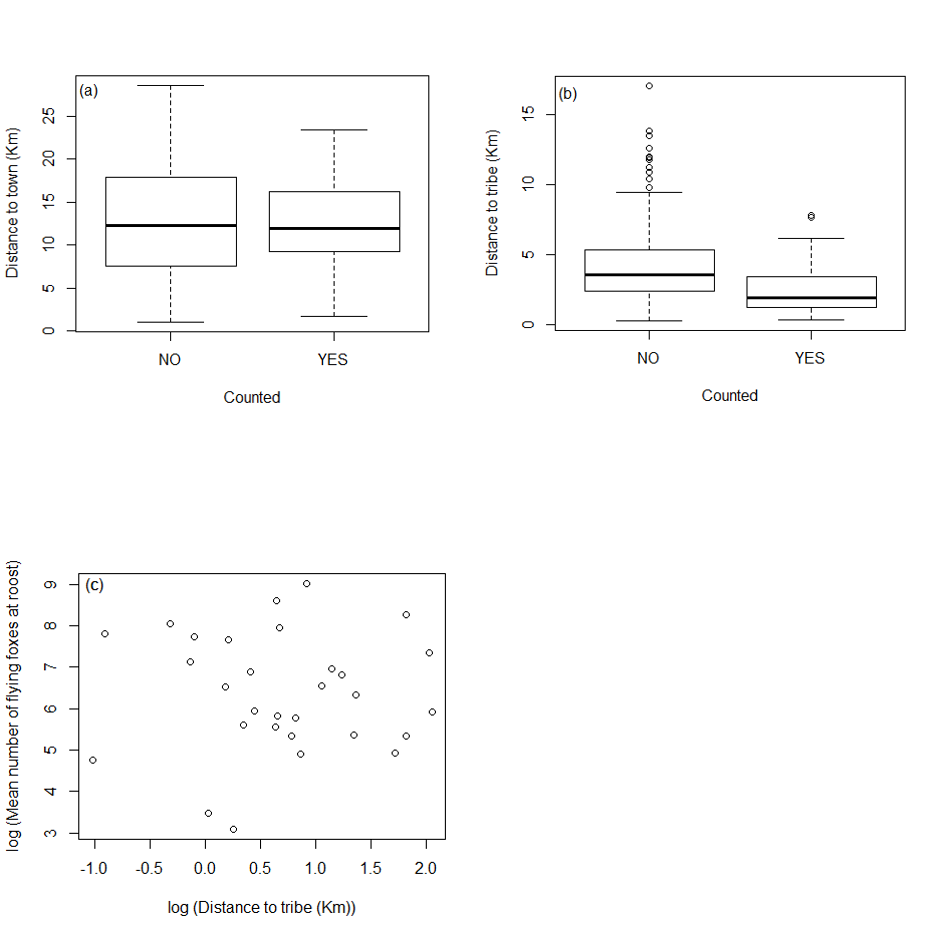

Supplement: S2 Fig — Distance to (a) the nearest town and (b) the nearest tribe according to whether the flying-fox roosts were part of the set of roosts selected for fly-out counts (counted: yes/no). (c) Correlation between the average size of roost and distance to the nearest tribe (both variables are log-10 transformed; r = 0.004, P = 0.99). (TIF) [file pone.0224466.s002.tif]
